# Transition models of care for type 1 diabetes: a systematic review

**DOI:** 10.1186/s12913-023-09644-9

**Published:** 2023-07-20

**Authors:** Yvonne Zurynski, Ann Carrigan, Isabelle Meulenbroeks, Mitchell N. Sarkies, Genevieve Dammery, Nicole Halim, Rebecca Lake, Elizabeth Davis, Timothy W. Jones, Jeffrey Braithwaite

**Affiliations:** 1 NHMRC Partnership Centre for Health System Sustainability, Australian Institute of Health Innovation, Macquarie University, Sydney, Australia; 2 Centre for Healthcare Resilience and Implementation Science, Australian Institute of Health Innovation, Macquarie University, 75 Talavera Road, North Ryde, Sydney, Australia; 3 Centre for Health Systems and Safety Research, Australian Institute of Health Innovation, Macquarie University, Sydney, Australia; 4 Telethon Kids Institute, University of Western Australia, Perth, Australia; 5 Perth Children’s Hospital, Perth, Australia

**Keywords:** Type 1 diabetes, Adolescents, Transition of care, Model of care

## Abstract

**Background:**

Managing the care regimen for Type 1 Diabetes is challenging for emerging adults, as they take on greater responsibility for self-management. A diverse range of models of care have been implemented to improve safety and quality of care during transition between paediatric and adult services. However, evidence about acceptability and effectiveness of these is limited. Our aim was to synthesise the evidence for transition models and their components, examine the health related and psychosocial outcomes, and to identify determinants associated with the implementation of person-centred models of transition care.

**Method:**

We searched Medline, CINAHL, EMBASE and Scopus. Peer reviewed empirical studies that focused on T1D models of care published from 2010 to 2021 in English, reporting experimental, qualitative, mixed methods, and observational studies were included.

**Results:**

Fourteen studies reported on health and psychosocial outcomes, and engagement with healthcare. Three key models of care emerged: structured transition education programs (6 studies), multidisciplinary team transition support (5 studies) and telehealth/virtual care (3 studies). Compared with usual practice, three of the six structured transition education programs led to improvements in maintenance of glycaemic control, psychological well-being, and engagement with health services. Four MDT transition care models reported improved health outcomes, and improved engagement with health services, however, three studies reported no benefit. Reduced diabetes related stress and increased patient satisfaction were reported by two studies, but three reported no benefit. Telehealth and virtual group appointments improved adherence to self-management and reduced diabetes distress but did not change health outcomes.

**Conclusions:**

Although some health and psychosocial benefits are reported, the results were mixed. No studies reported on T1D transition model implementation outcomes such as acceptability, adoption, and appropriateness among clinicians or managers implementing these models. This gap needs to be addressed to support future adoption of successful models.

**Supplementary Information:**

The online version contains supplementary material available at 10.1186/s12913-023-09644-9.

## Background

Type 1 diabetes (T1D) is a chronic and incurable autoimmune condition, typically diagnosed during childhood and managed initially in paediatric health care services until ages 16–18 years [1]. Paediatric diabetes care tends to be holistic, person- and family-centred, involving the family in care delivery and care planning. The focus is not only on medical management to ensure optimum glycaemic control, but also on the psychosocial adjustments of the child with T1D and their family [2]. Adult services tend to focus more on the patient as an individual rather than the family, and on self-management of routine diabetes care [2]. Visits to adult specialists tend to be shorter, more focused on medical issues, and each specialist is likely to be seen separately rather than the more holistic team-based approach in paediatric care [2]. The time of transition between paediatric and adult services can be difficult for all involved including the young person with T1D, their family and clinicians in both settings [3].

Emerging adults (EAs) with T1D face many challenges including juggling final years at school, coping with tertiary education or vocational training demands, changing personal relationships, new careers and other stressors, and their healthcare may be neglected [2, 4]. EAs may encounter barriers to accessing adult healthcare services, including a lack of age-appropriate information, arrangements or referral, reluctance of parents to relinquish control, or difficulties with transport or finances [4]. Virtual care and telehealth as key components of multidisciplinary, integrated care are showing promise to overcome some of the identified barriers, whilst offering convenience and flexibility, thereby improving the continuity of care [5].

The transition from paediatric to adult care is a crucial time for EAs as poorly controlled T1D can have lasting effects on their health and wellbeing, well into adulthood [6]. For EAs with T1D, erratic meal and exercise patterns are problematic [7], and treatment adherence rates reduce significantly leading to poor glycaemic control during and following transition from paediatric care [6]. There are higher rates of complications such as diabetic ketoacidosis and microvascular problems [6, 8], and lower clinic attendance rates are associated with these complications, suggesting sub-optimal care continuity crucial for ongoing management of their health care [6, 9–12]. In addition, EAs with T1D are more likely to experience depression [13], anxiety [14] and lower overall health-related quality of life at, or after, transition [15].

Traditionally, the transfer of care has occurred simply through a referral letter from the paediatric health professional to the adult health professional, but this has long been recognised as inadequate for successful continuity of care. According to the International Society for Pediatric and Adolescent Diabetes guidelines (ISPAD), the ideal time for counselling and preparation for transition is early puberty and the developing self-care capacity and confidence is supported when there is a trusting relationship between the EA and the diabetes care team who encourage self-reliance and self-efficacy [7]. Additionally, outcomes are improved when a parent(s) are involved in supporting the EA through transition, and when psychosocial issues are addressed early in preparation for transition [7].

The optimal care transition phase has been defined as a purposeful and planned process that prepares and builds capacity and skills for EAs to independently interact with adult health services and to undertake self-care activities [16, 17]. Increasingly, structured transition programs are being developed, implemented and accessed. Such programs bridge the gap across the paediatric-adult service divide and support EAs to ensure continuing engagement with health services whilst increasing skills for self-care [4]. A key aspect of transition is adequate time for preparation to ensure the EA, their family and health professionals in both settings are informed, skilled and ready [6].

Although there is an emerging body of literature describing T1D transition models of care, their components and outcomes are poorly understood [2]. For example, it is unclear what health and psychosocial benefits are associated with different models of transition. The implementation determinants of transition models of care for EAs with T1D, the barriers and enablers encountered when implementing these models into different clinical contexts and settings, are not known [2]. A systematic synthesis of the current evidence and knowledge about transition for T1D is needed to inform the development of future models of care or the enhancement and scaling up of existing models.

This review aims to synthesise the evidence for transition models of care, determine the model components, assess health related outcomes, and consider implementation determinants associated with person-centred models of care transition for EAs with T1D.

## Methods

### Review protocol

Our review was developed in accordance with the Preferred Reporting Items for.

Systematic Reviews and Meta-Analyses extension for Systematic Reviews (PRISMA) checklist [18]. This review follows a Prospero-registered protocol (CRD42021262727): https://www.crd.york.ac.uk/prospero/display_record.php?RecordID=262727.

### Search methods

The search strategy was designed in consultation with a medical librarian and the interdisciplinary review team. The search was executed on 6th June 2021 and updated on 20th November 2022 in four databases: Scopus, Medline, CINAHL, and EMBASE. Details of the search strategy for Medline is included in Box 1. and strategies for each database are described in Supplemental File 1. All searches were limited to publications in English, published from January 2010 to November 2022. To increase comprehensiveness, the search strategy also employed snowballing techniques, whereby the reference lists of included documents were searched for relevant publications and these additional publications were also screened according to the inclusion and exclusion criteria.

### Box 1. Search strategy for medline

Diabetes Mellitus, Type 1/ or diabetes mellitus/, (iddm or insulin dependent diabetes mellitus or insulin-dependent mellitus or type 1 diabetes or diabetes type 1).mp., 1 or 2, infant/ or child/ or adolescent/ or young adult/, (child* or infant* or teen* or adolescen* or young adult*).mp., 4 or 5, Clinical pathway/ or intervention study/ or evaluation study/, (model* of care or care model* or clinic* pathway* or referral pathway*).mp., (model* adj2 (service* or care)).ti,ab., "delivery of health care"/, (service* adj2 (initiativ* or configurat* or deliver* or capabilit*)).tw., (intervention* adj2 (target* or service* or strateg*)).tw., (service* adj2 (framework* or infrastructure)).tw., or/7-13, "delivery of health care, integrated"/ or transitional care/ or patient education/ or transition to adult care/ or Treatment Outcome/ or Outcome Assessment, Health Care/, (transitional care or care transitions or integrated care or multidisciplinary care or patient-centered care or transition to adult care or shared care plan or team-based care or team care or diabetes education or multidisciplinary team* or interdisciplinary care* or outcome*).mp., 15 or 16, 3 and 6 and 14 and 17.

### Inclusion and exclusion criteria

Peer-reviewed articles and literature reviews describing models of care implemented in an Organisation for Economic Co-operation and Development (OECD) Category 1 country [19] were included if they discussed an intervention targeted to patients under the age of 26 years with T1D and the intervention was a person-centred model of care. To be included, a study had to describe the model of care more broadly rather than simply discussing substitution of routine face-to-face consultations by telehealth. For example, we included studies that described multidisciplinary team (MDT) approaches facilitated by telehealth, or innovative diabetes education delivered via telehealth if these were embedded in a broader model of care where other model components were also described.

To be included, studies had to report on health or psychosocial outcomes, on satisfaction with the model of care at the patient, provider or parent/family level, and engagement with the adult health service. Studies reporting on implementation determinants including accessibility, acceptability, appropriateness, and satisfaction, from the perspective of the health consumer, caregiver and/or the healthcare provider were also included.

Studies were excluded if they were: published prior to 2010; published in a language other than English; conducted in a low- or middle-income country; or focused on non-transition care of T1D, Type 2 diabetes or maternal health interventions or clinical interventions (e.g., clinical trials involving drugs or specific equipment). Publications of opinion or perspective, commentaries, letters to the editor, editorials, and conference abstracts were also excluded. Studies solely describing delivering routine consultations through telehealth without a description of a broader model of care, were also excluded.

### Study selection

Reference details and abstracts for all returned searches were downloaded into an EndNote database and duplicates were removed. The deduplicated list was exported into the electronic screening program, Rayyan [20], where three reviewers (IM, MS, and YZ) independently screened titles and abstracts against inclusion/exclusion criteria. The review team met to discuss and develop a common understanding of the inclusion and exclusion criteria and how to apply them. Ten percent of articles were screened by IM and MS independently, and a separate sample of 10% was screened by YZ and IM. The same 10% of articles was screened by all three reviewers. Interrater Cohen’s kappa reliability scores were all above 0.6, which is considered a “good” inter-rater reliability score [21]. For the updated search, all title/abstracts and full texts were independently assessed by two reviewers (AC, RL). Disagreements among reviewers were resolved by discussion with the whole review team.

### Data extraction and synthesis

A custom data extraction workbook in Excel (Microsoft Corporation) was developed and pilot tested on five articles. Adjustments were made where necessary to fit the types of data reported in the articles. Data were systematically extracted by four reviewers (AC, MS, NH, RL). Any disagreements between reviewers were resolved via discussion. Key extracted information included study publication details (authors, year published); study setting, design and methods; patient details (age, sex, race/ethnicity, socio-economic status, mean duration of diabetes, health insurance status), model of care details (description of model components, staffing, resources, setting), description of usual care, health psychosocial or health service use outcomes, whether an implementation framework was used, and implementation determinants or enablers and barriers and adoption into practice were reported (Table 1). The data were analysed for common themes and features that comprised a specific model and categories of outcomes.

## Results

**Fig. 1:**
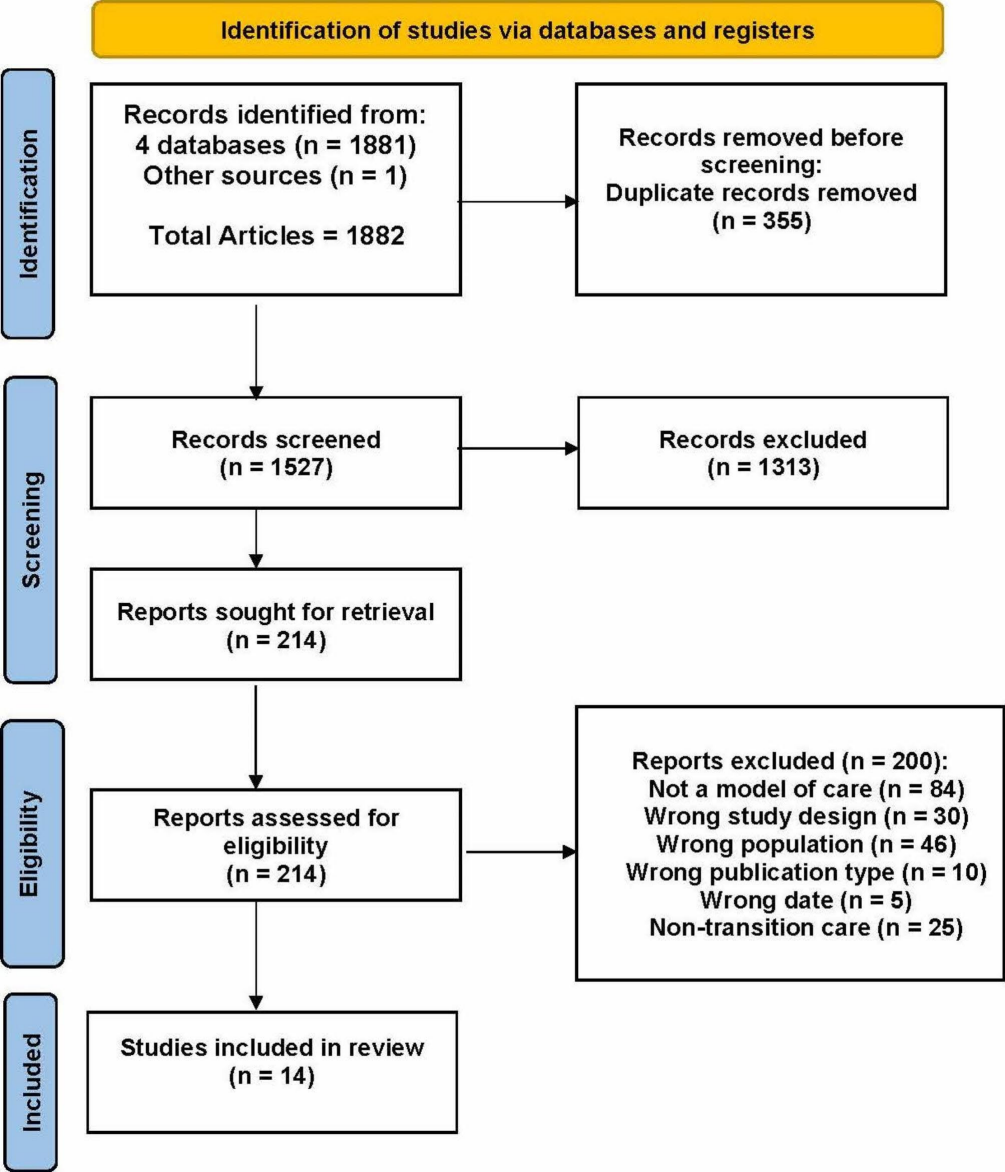
**PRISMA Flow Diagram of primary study selection**

The search for primary studies yielded 1882 results (CINAHL: 712, EMBASE: 572, Medline: 423, Scopus: 174; identified from other sources: 1). Among these, 355 duplicates were removed; after title/abstract screening, 1313 papers were excluded as they did not meet the inclusion criteria. Two hundred and fourteen studies underwent full-text review and a further 200 papers were excluded, leaving 14 included studies for data extraction and synthesis, (Fig. 1).

### Quality assessment

Studies were appraised using the Mixed Methods Appraisal Tool [22]. Two investigators (AC and RL) appraised 10% of the articles independently to ensure consistency. Quality assessment results were reported to reflect the quality of the studies included in our systematic review (Supplemental File 2). Nine of the 14 studies reported a quantitative non-randomised design, included a representative sample of participants, and used appropriate outcome measures [23–29]. However, one study did not present all the outcome data [25] and two did not account for confounders [25, 27]. There was only one randomised controlled trial that reported complete outcome data and adherence to the intervention, however, outcome assessors were not blinded, potentially introducing a bias [30]. There was one quantitative descriptive study [29], two mixed-methods studies [31, 32], and one qualitative methods study, all of which rated highly on the MMAT, (Supplemental File 2).We did not exclude any studies based on quality.

### The scope of transition models

Over half of the studies (8/14, 57%) were from the United States of America [23, 25–28, 30, 31, 33]. The remaining studies were from Australia (2/14, 14%) [29, 34], the United Kingdom (2/14, 14%) [24, 35], the Netherlands (1/14, 7%) [36], and Germany (1/14, 7%) [37], (Table 2). The models of care described in the 14 papers clustered around three main model types: (1) structured transition care program, (2) MDT transition support team, and (3) telehealth and virtual care as a component of a broader model (Table 1).

**Table 1:** Summary of the fourteen included studies

First author (reference no.)	Design	Country	Setting	Model of care implemented	Intervention duration and times at which outcomes were measured	Participants (n)	Age (mean years or range)**
Argarwal et al. 2017 [23]	Quantitative: retrospective cohort	USA	Metropolitan adult diabetes centre, connected to a university, and in partnership with a major children’s hospital	MDT transition support program, including paediatric partnership, care coordination, orientation to adult care, behavioural support, education, and enhanced engagement in care	6 months; Baseline and at 6 months	72	20.2
Bisno et al. 2021 [30]	Randomised control trial	USA	A multidisciplinary metropolitan diabetes clinic, within a university medical school	Telehealth, involving three regular telehealth appointments with a diabetes specialist, and one in person appointment per year; four virtual group appointments with other young adults with T1D	12 months; Baseline and 12 months	58	20.5
Colver et al. 2018 [24]	Quantitative: longitudinal	UK	Five paediatric diabetes centres, NHS trusts	MDT transition support features, including coordinated team, age banded clinic, life skills training, promotion of health efficacy, meeting the adult care team before transition, healthy parental involvement, written transition plan, key support person, and transition manager	4 years; Annually for study duration	150	14 -18.9
Egan et al. 2015 [31]	Mixed methods: prospective longitudinal	USA	Large metropolitan university hospital, including paediatric and adult diabetes centres	MDT transition support, involving a joint meeting between paediatric and adult care teams with the patient	Ongoing; Baseline, 3–6 months post transition and 12 months post transition	29	21
Farrell et al. 2018 [29]	Quantitative: retrospective descriptive	Australia	Outpatient clinic within a major metropolitan public hospital	MDT transition support, including ensuring first appointment is less than six months from the last paediatric appointment, SMS appointment reminders, rebooking of missed appointments, a central phone contact, a diabetes educator as clinic coordinator, late closing hours, MDT care team, and phone support	Service evaluation (ongoing); 18 months and 30 months following transition	684	18
Lyons et al. 2021 [25]	Quantitative: longitudinal	USA	Four paediatric endocrinology centres, and one adult practice, based within metropolitan hospitals or diabetes centres within universities	Quality improvement programs; sites developed their own QI programs, focusing on measures to educate patients about insulin pump usage, and support them in starting and continuing pump usage	15 months; Baseline, monthly during intervention (15 months) and post-intervention (2 months)	aggregated data in each site	12–26
Peeters et al. 2021 [36]	Mixed methods: retrospective	Netherlands	MDT paediatric and adult diabetes care teams at twelve hospitals	MDT transition support; teams were clustered into groups based on whether they paid high or low attention to transitions	2 years; One- and two-years post transfer	320	23.7
Price et al. 2011 [35]	Qualitative	UK	Diabetes clinic within one NHS general hospital	Structured transition program; interviews identified two super-ordinate themes among young adults—transition services should be developmentally appropriate and based around individual needs	6 months; Baseline, 3 and 6 months	11	16–18
Pyatak et al. 2016 [26]	Quantitative: longitudinal	USA	Metropolitan paediatric diabetes centres, emergency departments, community health centres, primary health clinics	Comparison of patients in final year of paediatric care and receiving continuous care, with those lost to care following unsuccessful transition	12 months; Baseline, 6 and 12 months	75	20
Raymond et al. 2016 [27]	Quantitative: pilot cross sectional	USA	Metropolitan primary care clinic	Telehealth, involving a virtual clinic visit and a virtual group appointment with other young adults with T1D	Not stated	45	20
Reid et al. 2018 [28]	Quantitative: prospective	USA	A multidisciplinary metropolitan diabetes clinic within a university medical school	Telehealth, involving three regular telehealth appointments with a diabetes specialist, and one in-person appointment per year	9 months; Baseline, 3,6 and 9 months	81	19.8
Rueter et al. 2021 [34]	Quantitative: retrospective	Australia	Transition clinic at a metropolitan public hospital, as well as adult public hospital and clinic visits recorded by the ADDN and ANDA	MDT transition support clinic, involving three monthly appointments and appointment re-booking, dedicated transition coordinator/educator, complication screening, and tailored clinic hours	18 months	1,604	20.3
Schmidt et al. 2018 [37]	Quantitative: cross sectional	Germany	Various health centres including mainly paediatric sub-specialty clinics in tertiary care hospitals, and one inpatient rehabilitation centre	Structured transition education program, consisting of a two-day transition workshop containing eight modules	6 months; Baseline and 6 months	153	16.4
Sequeria et al. 2015 [33]	Quantitative: longitudinal	USA	Three paediatric diabetes clinics within major urban hospitals	Structured transition program, including education, group education, case management, and access to a new resource website	12 months	81	19–25

MDT: multidisciplinary team; NHS: National Health Service, United Kingdom; SMS: short message service; ADDN: Australian Diabetes Data Network; ANDA: Australian National Diabetes Audit

### Model components

**Fig. 2:**
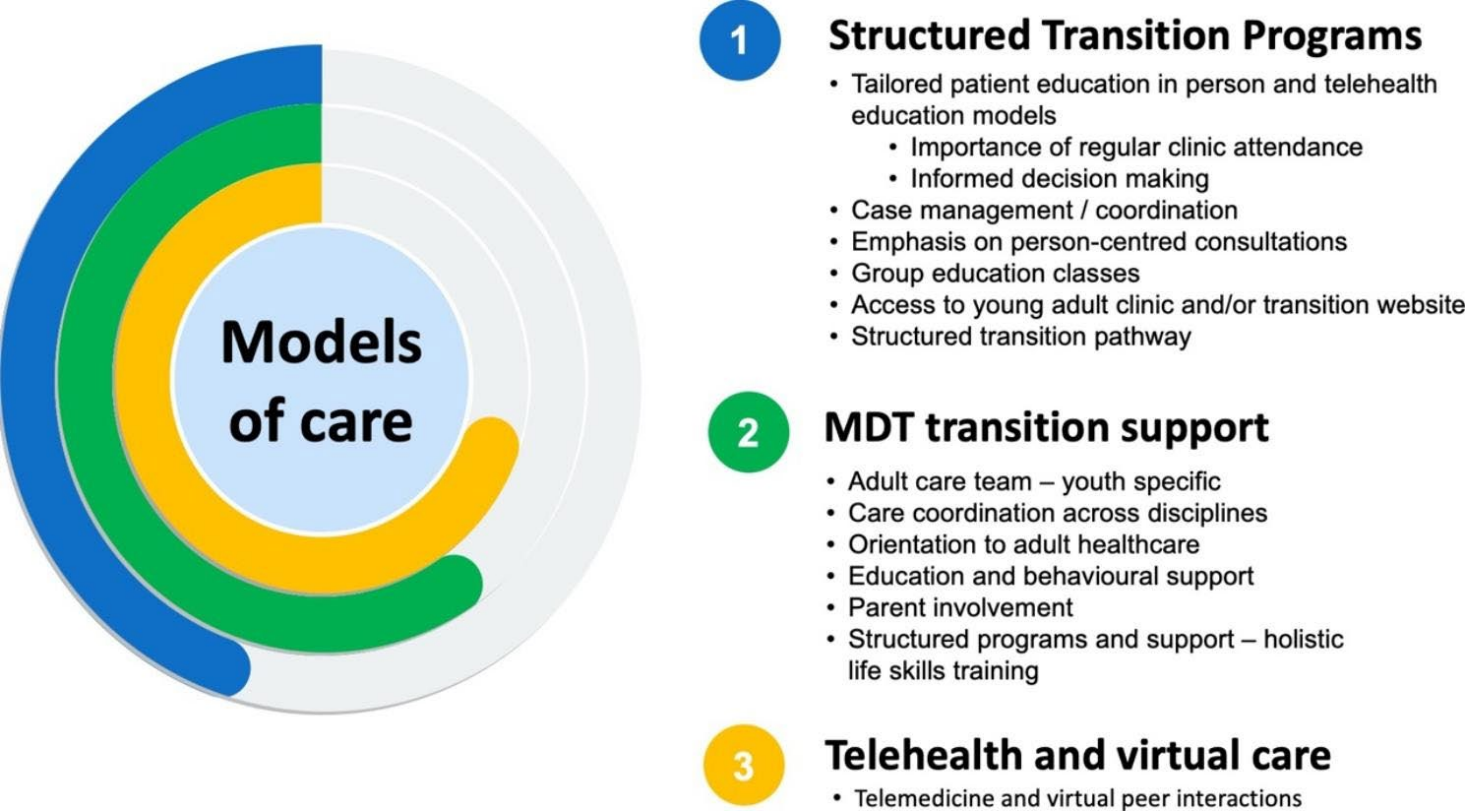
Models of care and their components

Model components included MDT care where the paediatric team and adult team worked together; structured preparation and educational programs or modules for EAs; involvement of parents or primary caregivers in the transition process; group support sessions with peers or group educational programs for EAs (in person or on-line); joint appointment(s) involving the paediatric and adult endocrinologists; detailed transition plans shared with providers and the EAs (although this was done to varying degrees); involvement of a coordinator, navigator or case manager; and telehealth consultations. Three broad types of models of care were identified that included a diverse variety of the above components (Fig. 2).

### Structured transition care program

A structured transition care program was reported in six (43%) of the 14 included studies [25, 26, 33, 35–37]. These programs included a range of structured services for the patient involving active preparation and skills development for self-care, case management and access to online resources.

### MDT transition support

A MDT transition support team was reported in five (36%) of the 14 included studies [23, 24, 29, 31, 34]. MDTs included a variety of healthcare providers, for example, endocrinologists, psychologists, nurses, diabetes educators, social workers and dieticians, and in some cases healthcare providers from paediatric and adult services working together [23, 24, 31].

### Telehealth and virtual care as a component of a broader model

Telehealth and virtual care were reported in three of the 14 included studies [27, 28, 30]. This model involved telehealth with a diabetes clinician and/or diabetes educator, and virtual, peer support groups.

### Health and psychosocial outcomes

The wide variety of outcome measures used across the different studies (Table 2), made synthesis of evidence challenging.

**Table 2:** Examples of outcomes measures reported

Outcome	Examples
Health	Glycaemic control, HbA1c levels, diabetic ketoacidosis, insulin pump usage, blood glucose monitoring frequency, adherence to routine visits, unscheduled presentations to emergency services.
Psychosocial	Diabetes-related distress, depression, wellbeing, stress, quality of life, self-management and transition preparedness, diabetes empowerment, life satisfaction, problem-solving skills and communication with health professionals.
Satisfaction with model	Patient, provider and parent satisfaction with the models of care, and satisfaction with health services or appointments.
Engagement with adult health services	Clinic visits, participation in appointments, care adherence.
Other	Transfer competence, diabetes knowledge, length of hospital stay, patient experience with transition, time convenience.

### Structured transition care programs

The outcomes reported in the six studies that described structured transition care programs, were varied and some showed benefits whilst others showed no change (Table 3). Four studies reported health outcomes: two of these reported improvements in glycaemic control [26, 33] and one reported no change [36]. One study reported an increase in insulin pump usage [25]. All six studies reported on psychosocial outcomes. There were improvements in well-being and reduced stress [33], and improved life satisfaction [26]. Other reported benefits included greater engagement with adult services with an increase in post-transition clinic visits [36], increased diabetes knowledge [26, 33], improved transfer competence [37], and positive patient experiences with the transition process [35], (Table 3). However other studies reported no change in quality of life [37], depression [26], diabetes empowerment or life satisfaction [33].

### MDT transition support programs

The effectiveness of MDT models was also mixed (Table 3). Four studies reported benefits related to glycaemic control [23, 29] and diabetic ketoacidosis [29, 34], however, two studies reported no benefits for glycaemic control [31, 34]. Two studies reported benefits for blood glucose monitoring frequency [23] and insulin pump usage [29].

One of two studies that reported on psychosocial outcomes, reported benefits for wellbeing [24] and the other showed benefits in terms of reduced diabetes distress [31], however, the second study reported no benefit for quality of life [31]. Increased satisfaction with the model of care was reported for patients [24, 31], but this was not consistent across studies [23]. There was some evidence for improved healthcare provider satisfaction [23], but no benefits were reported for parent satisfaction in the one study that measured this outcome [31].

Keeping scheduled appointments at adult clinics and participation in the consultations had increased [24, 29, 34], and one study reported reduced length of hospital stays associated with an MDT model [29], (Table 3).

### Telehealth and virtual care as a component of a broader model

Three included studies were based on one virtual care model, the Colorado Young Adults with Type 1 Diabetes (CoYoT1), which incorporated telehealth for clinic visits and virtual peer group appointments with a diabetes educator. The pilot feasibility study of CoYoT1, reported high levels of patient satisfaction and an average of six hours travel time saved when attending their clinic online rather than in person, due to less travel [27]. The second CoYoT1 study showed increased clinic attendance rates that met the American Diabetes Associations’ guidelines, higher appointment satisfaction, with no reduction in HbA1c values, compared with usual care [28]. The third CoYoT1 study was a randomised controlled trial that compared two care delivery modes, one combined telehealth and virtual group appointments, and the other used telehealth alone. There were no differences in HbA1c, use of continuous glucose monitors or insulin pumps, quality of life, depression, problem-solving skills or communication with carers. However, the combination of and telehealth and virtual group appointments was associated with decreased diabetes distress [30], (Table 3).

**Table 3:** Evidence for effectiveness of models of care stratified by outcome type

Model	Outcome	Variable measured	First author (reference no.	Evidence	Benefit?
Structured transition program					
	Health	Insulin pump usage	Lyons et al. 2021 [25]	Increased in-person and telehealth education about insulin pump technology resulted in 13% increased usage of insulin pump (45–58%) over a 22-month period	Benefit
		Glycaemic control	Peeters et al. 2021 [36]	No difference in mean HbA1c levels between those who received more attention about their care, compared with those who received less attention. Only 10.6% reached their targeted scores	No change
		Glycaemic control	Pyatak et al. 2016 [26]	Compared with lapsed care, continued care had lower levels of HbA1c and a reduction in severe hypoglycaemia and associated ED admissions at 12 months	Benefit
		Glycaemic control	Sequeria et al. 2015 [33]	Compared with usual care, the intervention group who received a structured transition program had significant improvements in glycemic control and a lower incidence of severe hypoglycaemia at 12 months	Benefit
	Psychosocial	Self-management and transition preparedness	Peeters et al. 2021 [36]	There was no difference in self-management between those who received more attention about their care compared with those who received less attention. The more attention group felt better prepared for transfer, compared to the less attention group	No change in self-management. Benefit for transition preparedness
		Quality of life	Schmidt et al. 2018 [37]	No effect of a two-day patient education program on quality of life	No change
		Depression	Pyatak et al. 2016 [26]	Compared with continual care, levels of depression and perceived stress for those who experience lapsed care did not improve after 12 months	No change
		Well-being and stress	Sequeira et al. 2015 [33]	Compared with a control group, the intervention group reported an improvement in global well-being and perceived stress after 12 months	Benefit
		Diabetes empowerment	Sequeira et al. 2015 [33]	Compared with pre intervention, there were no significant changes in diabetes empowerment	No change
		Life satisfaction	Sequeira et al. 2015 [33]	Compared with pre intervention, there were no significant changes in life satisfaction.	No change
		Life satisfaction	Pyatak et al. 2016 [26]	Those who received continual care reported higher levels of overall life satisfaction compared with lapsed care	Benefit
	Engagement with adult health services	Number of post-transition clinic visits	Peeters et al. 2021 [36]	High attention group scheduled more consultations in the year after transfer of care compared with the low attention group	Benefit
	Other	Transfer competence	Schmidt et al. 2018 [37]	A positive effect on health-related transition competence after a two-day patient education program	Benefit
		Diabetes knowledge	Sequiera et al. 2015 [33]	There was no difference in diabetes knowledge for the intervention group compared with a control group	No change
		Diabetes knowledge	Pyatak et al. 2016 [26]	At 12 months, those who received continual care reported higher levels of diabetes knowledge compared with lapsed care	Benefit
		Patient experience	Price et al. 2011 [35]	Essential that services are designed to be developmentally appropriate and consumer focused, and the consultation experience is paramount in facilitation of healthcare service engagement	Benefit
MDT transition support					
	Health	Glycaemic control	Agarwal et al. 2017 [23]	Mean A1C reduced from 9.7–9% (p < 0.001) across a 6-month period	Benefit
		Glycaemic control	Egan et al. 2015 [31]	There was no difference in HbA1C levels between pre- and post-transition (8.7% − 8.4%). Higher levels of diabetes-related distress were associated with higher HbA1C levels	No change
		Glycaemic control	Farrell et al. 2018 [29]	The continuity of care post-transition prevented deterioration in HbA1c. Compared with those who did not attend the MDT clinic, attendees had lower baseline HbA1c levels at 18-month and 30-month follow up No change in HbA1c between the first and 30-month follow up appointments	Benefit
		Glycaemic control	Rueter et al. 2021 [34]	Compared with data registries, there was no change in HbA1c levels for a transition clinic. No impact of socio-economic status on glycaemic control	No change
		Diabetic ketoacidosis	Farrell et al. 2018 [29]	Admissions for diabetic ketoacidosis were reduced with age-appropriate education and regular follow-up	Benefit
		Diabetic ketoacidosis	Rueter et al. 2021 [34]	Diabetic ketoacidosis admissions were significantly reduced for increased clinic attendance	Benefit
	Adherence	Blood glucose monitoring frequency	Agarwal et al. 2017 [23]	Blood glucose monitoring frequency increased by one check per day from 2.5 to 3.5 (p < 0.001)	Benefit
		Insulin pump usage	Farrell et al. 2018 [29]	Data from 11 years showed a significant increase in pump usage from 0–40%	Benefit
	Psychosocial	Quality of life	Egan et al. 2015 [31]	Higher levels of HbA1c were strongly associated with lower quality of life. No change in quality of life between pre- and post-transition. A strong, positive correlation was seen between diabetes-related distress and quality of life	No change
		Wellbeing	Colver et al. 2018 [24]	Parent involvement was positively associated with wellbeing (p < 0.0001)	Benefit
		Patient diabetes-related distress	Egan et al. 2015 [31]	Significantly reduced distress between pre- and post-transition for patients (p = 0.021) and their parents (p = 0.012)	Benefit
	Satisfaction	Patient satisfaction	Agarwal et al. 2017 [23]	Positive responses for model acceptance, ease of transfer and having a dedicated adult care team. Patients reported feeling informed and motivated as the clinic focused on individual needs. Negative responses about travel issues for some	Mixed
		Patient satisfaction	Egan et al. 2015 [31]	Most patients felt satisfied with the program and the degree to which providers supported their autonomy. Many noted that they enjoyed being in a more adult venue, they felt more in charge of their care, and were more informed about complications	Benefit
		Parent satisfaction	Egan et al. 2015 [31]	Parents perceived their emerging adult as ready for transition, but they themselves were not. They described not having enough information prior to transition. All parents continued to be involved in care processes such as managing health insurance	No change
		Provider satisfaction	Agarwal et al. 2017 [23]	Positive responses for adult and paediatric providers	Benefit
		Patient satisfaction of health service	Colver et al. 2018 [24]	Patient satisfaction of health service was positively associated with health self-efficacy (p = 0.006)	Benefit
	Engagement with adult health services	Participation in appointments	Colver et al. 2018 [24]	Meeting the adult team prior to transfer was positively associated with participation (p < 0.0001) and autonomy in appointments (p < 0.0001)	Benefit
		Number of clinic visits	Farrell et al. 2018 [29]	Compared with those who did not attend the MDT clinic, attendees had higher cumulative clinic visits at 18-month and 30-month follow up	Benefit
		Interval between clinic visits	Reuter et al. 2021 [34]	Compared with data registries, median interval between clinic visits was shorter for MDT transition clinic attendees	Benefit
	Other	Length of hospital stay	Farrell et al. 2018 [29]	Time spent in hospital was significantly reduced for those attending the MDT clinic	Benefit
Telehealth and Virtual Care					
	Health	Glycaemic control	Reid et al. 2018 [28]	Number of blood glucose checks and HbA1c values did not change over the nine months	No change
		Glycaemic control	Bisno et al. 2018 [30]	No difference in HbA1c levels between intervention and control group over twelve months (p = 0.6)	No change
		Continuous glucose monitor use	Bisno et al. 2018 [30]	No difference between intervention and control group over twelve months (p = 0.53)	No change
		Insulin pump use	Bisno et al. 2018 [30]	No difference between intervention and control group over twelve months (p = 0.63)	No change
	Psychosocial	Quality of life	Bisno et al. 2021 [30]	No difference between intervention and control group over twelve months (p = 0.89)	No change
		Depression	Bisno et al. 2021 [30]	No difference between intervention and control group over twelve months (p = 0 0.71)	No change
		Diabetes distress	Bisno et al. 2021 [30]	Intervention group reported lower levels of T1D-related distress on average than control group (p = 0.02)	Benefit
		Self-perceived ability	Bisno et al. 2021 [30]	No differences in self-confidence, ability to manage symptoms, or self-efficacy (p > 0.05)	No change
	Satisfaction	Satisfaction with clinic	Raymond et al. 2016 [27]	Patients reported high levels of satisfaction with the clinic	Benefit
		Appointment satisfaction	Reid et al. 2018 [28]	Intervention group reported high levels of appointment satisfaction across six months than controls (p = 0.03)	
	Engagement with adult health services	Clinic attendance	Reid et al. 2018 [28]	Compared with controls, intervention group attended more clinic visits (p < 0.0001)	Benefit
		Care adherence	Reid et al. 2018 [28]	Intervention group adhered to care recommendations at approximately twice the rate of controls	Benefit
	Other	Communication with carers	Bisno et al. 2021 [30]	No difference in communication with care providers about symptoms and care between intervention and control group over twelve months (p = 0.07)	No change
		Problem-solving skills	Bisno et al. 2021 [30]	No difference in problem-solving abilities between intervention and control group over twelve months (p = 0.051)	No change
		Time convenience	Raymond et al. 2016 [27]	A saving of over six hours from their day when completing their clinic virtually compared with usual care	Benefit

ED: Emergency Department; MDT: multidisciplinary team.

### Implementation determinants of transitional models of care

None of the fourteen studies assessed implementation strategies or drivers of model adoption, and none mentioned implementation frameworks or theories. However, most studies discussed the enablers, and some reported on the barriers for the implementation of identified models, (Table 4).

**Table 4:** Enablers and barriers of implementation

Overarching model	Model description	First author (reference no.)	Enablers	Barriers
MDT transition support	The paediatric to adult diabetes transition clinic	Agarwal et al. 2017 [23]	Program was developed inside of an existing adult diabetes clinic, which already had resources. There was an established relationship with the hospital that facilitated referrals. Model was preferred as it was adult health care based and developmentally appropriate	Nil reported
MDT transition support	Four home visits to present proposed beneficial features associated with transfer of care	Colver et al. 2018 [24]	Appropriate level of parental involvement as the EA takes on responsibilities for self-care, promotion of health self-efficacy, and meeting with the adult team before transfer	Nil reported
MDT transition support	Joint meetings between the paediatric diabetes care providers, and the adult team, along with educators, nurse practitioner and the young adult	Egan et al. 2015 [31]	Evening appointments, a coordinated and collaborative effort by paediatric and adult programs, MDT presence at joint meetings the provision of a concrete timeline for transition with a plan, a transition coordinator, and a sense of partnership between paediatric and adult health care teams	Parental lack of preparation and knowledge about the transfer of care
MDT transition support	Care provided by a diabetes specialist, primary care physician and diabetes educator, supported by a transition coordinator	Farrell et al. 2018 [29]	Appointment reminders and active rebooking of missed appointments, and regular follow-up on sick day management. A collaborative relationship with paediatric service, promoting early engagement with the adult service	Travel distance for some as service based in a metropolitan centre
Structured transition program	Plan-Do-Study-Act cycles: Quality improvement programs focusing on measures to educate and support patients about insulin pump usage	Lyons et al. 2021 [25]	Improved patient education and support, cooperative culture, engagement with staff, sustainment of visits and increasing frequency of touchpoints	Barriers to using the pump include cost/unaffordability, personal preference, and lack of familiarity with technology
Structured transition program	“On your own feet” transition care framework	Peeters et al. 2021 [36]	Parental involvement, knowledge and skills brought about by more frequent consultations around transition; access to transition coordinator to “bridge the gap” between settings	Lack of structured support for parents
Structured transition program	Transition pathway that is developmentally appropriate and based around individual needs	Price et al. 2011 [35]	Training for professionals delivering the service, that included communication skills, and the importance of a person-centred care approach	Nil reported
Structured transition program	Tailored education, case management, group education classes, access to adult clinic and transition website	Sequeria et al. 2015 [33]	Program structure, team approach, availability of a case manager at both discharging paediatric clinic and accepting adult clinic	Nil reported
Telehealth and Virtual Care	Colorado Young Adults with T1D (CoYoT1) Virtual Clinic	Raymond et al. 2016 [27]	High levels of digital literacy among participants. Technology enabled flexibility of access to care	Nil reported
	Colorado Young Adults with T1D (CoYoT1) Virtual Clinic	Reid et al. 2018 [28]	Technology enabled flexibility of access	Internet firewalls at work for EA patients
	Colorado Young Adults with T1D (CoYoT1) Virtual Clinic	Bisno et al. 2021 [30]	It was perceived that telehealth improved communication with care providers	Nil reported

MDT: multidisciplinary team

## Discussion

Our results address a gap in knowledge about the nature, acceptability, and effectiveness of implemented models of care that support EAs with T1D transitioning from paediatric to adult services. However, many gaps in knowledge remain because of the limited number of studies, and the wide variability of models of care, model components, and outcome measures being reported. Our synthesis identified three emerging models of care that have been implemented to support transition including (1) structured transition care programs, (2) MDT transition support, and (3) telehealth or virtual care embedded as part of a broader model.

The evidence of effectiveness of structured transition care programs was mixed. Some studies reported positive health [25, 26, 33], and psychosocial outcomes [33], life satisfaction [26], and diabetes knowledge [26], while other studies reported no effect. In one study, the availability of a case manager at the time of transition enabled these positive health and psychosocial outcomes [33]. Other studies found no effect on self-management [36], quality of life [37], depression and stress [26], and diabetes knowledge [33]. Some of these mixed findings could be explained by study design and the wide variety of included model components within the three broad model types emerging from the literature.

Of the five studies describing MDT transition support including an adult care team in addition to the paediatric team, care coordination, parental involvement, and structured programs, some found positive benefits for glycaemic control [23, 29], reduced diabetic ketoacidosis [34], reduced time spent in hospital [29], better adherence to clinic visits [34], and improved wellbeing with the right level of parent involvement [24], although the “right level” of parent involvement was seldom clearly defined. Patient and parent satisfaction of the MDT transition support models was highly rated in two studies [23, 31] but parent satisfaction did not increase [31]. Meeting the adult team and the supported integration of EAs into the adult service was feasible and acceptable to EAs [24]. These findings are consistent with the 2018 International Society for Pediatric and Adolescent Diabetes guidelines which suggest that a supportive team that includes paediatric and adult care clinicians, with the involvement of parents leads to better care during transition and better health outcomes [7].

Healthcare providers’ perceptions of the value, acceptability and feasibility of innovative models of care delivery have been widely recognised as important to model implementation and sustainability [38]. However, only one study measured provider satisfaction [23]. Understanding health care provider views is important to inform service planning, staff capacity building and upskilling, and for the future development, implementation at scale, and evaluation of models of care [39, 40]. The limited information on the views of clinicians, educators and managers involved in the implementation and delivery of transitional models of care is a significant gap in the current literature.

Three studies based on the CoYoT1 model showed the value of supporting EAs with T1D through telehealth and virtual group appointments [27, 28, 30]. Diabetes distress decreased, quality of life and problem-solving skills improved, as did communications between EAs and health professionals [30]. EAs participating in CoYoT1, reported high levels of satisfaction because of flexibility, convenience, improved access and engagement with the adult clinical team [27, 28, 30]. High levels of digital literacy among EAs was recognised as an important enabler for this model [27]. These findings are consistent with other literature that reports the link between digital health technologies and digital health literacy and greater engagement with, and access to, health services [41].

The sustainability of the innovative models of care described in this literature review cannot be assessed, mainly because outcomes were mostly assessed over short follow-up time frames. Twelve studies reported outcomes at 6–12 months and only two studies in this review, Farrell et al. 2018 [29] and Peeters et al. 2021 [36], measured outcomes two years or longer after implementation, suggesting some level of sustainability, although sustainability was not explicitly assessed [42].

Across the three types of models of care, the key reported attributes for successful transition included building positive relationships, patient-centred education, and integration into the adult clinics supported by an MDT approach to care. Technology enablers including telehealth, apps and web-based peer support groups as well as flexible access to a case manager or coordinator that works with the EAs and clinical teams to smooth the transition journey according to the EA’s individual health and psychosocial needs and capabilities, were all considered important enablers. The importance of transition programs that include coordinators or navigators has been discussed and recommended for many years, however, such models of care are not widely implemented [43]. Notable successful examples include the Trapeze Transition care program in New South Wales Australia [44], and the Transition to Adult Care (On TRAC) program in British Columbia, Canada [45]. These transition programs are not disease specific and aim to assist EAs with many different chronic conditions. There appears to be untapped potential to learn from and leverage such programs when supporting EAs living with T1D to transition successfully to adult care [43]. Most studies included in this review focused on the positive aspects of the model they were reporting on, and mentioned barriers less frequently. Barriers associated with the transition models of care included parental lack of preparation and knowledge [31], long travel distances for patients and families to access transition care [29], and financial burden among those required to use insulin pumps [25]. Limited digital literacy or access to the internet impacted the effective use of for telehealth or virtual care and limited training and technological skills impacted the use of continuous glucose monitors and insulin pumps [25, 28]. These factors should be carefully considered when co-designing, co-producing or scaling up models of transitional care for EAs with T1D.

Understanding the factors influencing implementation of transitional models of care is crucial for improving care for EAs with T1D and for future implementation of successful models at scale. No study in the current review addressed implementation drivers at the provider or health system level. Future studies should examine implementation outcomes of T1D transition models including levels of acceptability, adoption, appropriateness, fidelity, penetration into the healthcare system, and cost, by utilising a framework such as the Consolidated Framework for Implementation Research (CFIR) [46]. The CFIR is an apt example of an organising framework that provides a guide for systematically assessing potential barriers and facilitators for implementation and to guide implementation planning and evaluation [47]. To support future implementation and evaluation of transitional models of care, understanding readiness for implementation at the individual, team and organisational level is also an important consideration [48], however, none of the studies included in our review touched on these aspects.

### Strengths and limitations

A comprehensive search and rigorous study selection strategy was used to identify relevant studies from a range of academic databases. However, limiting the search only to articles written in English, is likely to have omitted relevant evidence written in other languages. We were not able to pool data due to the wide heterogeneity of study methodologies, analysis methods, and outcome measures. The generalisability of our findings is limited by study designs used (e.g., retrospective designs or lack of comparator group), and a lack of perspective from the providers’ who deliver care under these new models. The variable inclusion of model components and high variability of the characteristics of model components made it challenging to classify the models of care into cohesive groups.

## Conclusion

Across three broad transition model types identified in this review, reported benefits for transitioning EAs with T1D include improved health outcomes such as glycaemic control, better engagement with the health system in terms of attendance at regular appointments, reduced presentations to emergency departments and reduced diabetes-related stress, although not all studies reported these benefits. We identify a need to improve the scope and quality of current evidence, which was based on only 14 studies with mostly small sample sizes, and limited follow-up periods. Economic analyses and analyses of acceptability, adoption, appropriateness, and feasibility at the level of clinical teams, funders, and managers were rarely reported. The body of evidence needs to be strengthened through rigorously designed studies that are guided by implementation frameworks, to better understand barriers, enablers and drivers of model effectiveness, acceptability, adoption and sustainability.

## Electronic supplementary material

Below is the link to the electronic supplementary material.

Supplementary Material 1

Supplementary Material 2

## Data Availability

The datasets used and/or analysed during the current study are available from the corresponding author on reasonable request.

## References

[1] Busse F, Hiermann P, Galler A, Stumvoll M, Wiessner T, Kiess W (2007). Evaluation of patients’ opinion and metabolic control after transfer of young adults with type 1 diabetes from a pediatric diabetes clinic to adult care. Horm Res Paediatr.

[2] Peters A, Laffel L, Group ADATW. Diabetes care for emerging adults: recommendations for transition from pediatric to adult diabetes care systems: a position statement of the American diabetes association, with representation by the American College of osteopathic family physicians, the American Academy of pediatrics, the American association of clinical endocrinologists, the American osteopathic association, the centers for disease control and prevention, children with diabetes, the endocrine Society, the International Society for pediatric and adolescent diabetes, juvenile diabetes research Foundation international, the National diabetes education program, and the pediatric endocrine Society (formerly Lawson Wilkins pediatric endocrine Society). Diabetes Care. 2011;34(11):2477-85. 10.2337/dc11-1723 PMC3198284 22025785

[3] Garvey KC, Markowitz JT, Laffel L (2012). Transition to adult care for youth with type 1 diabetes. Curr Diabetes Rep.

[4] Jones SE, Hamilton S (2008). The missing link: paediatric to adult transition in diabetes services. Br J Nurs.

[5] Smith AC, Thomas E, Snoswell CL, Haydon H, Mehrotra A, Clemensen J (2020). Telehealth for global emergencies: implications for coronavirus disease 2019 (COVID-19). J Telemed Telecare.

[6] Lotstein DS, Seid M, Klingensmith G, Case D, Lawrence JM, Pihoker C (2013). Transition from pediatric to adult care for youth diagnosed with type 1 diabetes in adolescence. Pediatrics.

[7] Cameron FJ, Garvey K, Hood KK, Acerini CL, Codner E (2018). ISPAD clinical practice consensus guidelines 2018: diabetes in adolescence. Pediatr Diabetes.

[8] Burns K, Farrell K, Myszka R, Park K, Holmes-Walker DJ (2018). Access to a youth‐specific service for young adults with type 1 diabetes mellitus is associated with decreased hospital length of stay for diabetic ketoacidosis. J Intern Med.

[9] Bryden KS, Dunger DB, Mayou RA, Peveler RC, Neil HAW (2003). Poor prognosis of young adults with type 1 diabetes: a longitudinal study. Diabetes Care.

[10] Garvey KC, Wolpert HA, Rhodes ET, Laffel LM, Kleinman K, Beste MG (2012). Health care transition in patients with type 1 diabetes: young adult experiences and relationship to glycemic control. Diabetes Care.

[11] Kipps S, Bahu T, Ong K, Ackland F, Brown R, Fox C (2002). Current methods of transfer of young people with type 1 diabetes to adult services. Diabet Med.

[12] Spaic T, Robinson T, Goldbloom E, Gallego P, Hramiak I, Lawson ML (2019). Closing the gap: results of the multicenter canadian randomized controlled trial of structured transition in young adults with type 1 diabetes. Diabetes Care.

[13] Pinquart M, Shen Y (2011). Depressive symptoms in children and adolescents with chronic physical illness: an updated meta-analysis. J Pediatr Psychol.

[14] Brady AM, Deighton J, Stansfeld S (2017). Psychiatric outcomes associated with chronic illness in adolescence: a systematic review. J Adolesc.

[15] Varni JW, Limbers CA, Burwinkle TM (2007). Impaired health-related quality of life in children and adolescents with chronic conditions: a comparative analysis of 10 disease clusters and 33 disease categories/severities utilizing the PedsQL™ 4.0 generic core scales. Health Qual Life Outcomes.

[16] Sawyer S, Blair S, Bowes G (1997). Chronic illness in adolescents: transfer or transition to adult services?. J Paediatr Child Health.

[17] Marani H, Fujioka J, Tabatabavakili S, Bollegala N (2020). Systematic narrative review of pediatric-to-adult care transition models for youth with pediatric-onset chronic conditions. Child Youth Serv Rev.

[18] Page MJ, McKenzie JE, Bossuyt PM, Boutron I, Hoffmann TC, Mulrow CD (2021). The PRISMA 2020 statement: an updated guideline for reporting systematic reviews. Int J Surg.

[19] Organization for Economic Co-operation and Development. Country Classification 2021 [Available from: https://www.oecd.org/trade/topics/export-credits/documents/2021-cty-class-en-(valid-from-18-08-2021).pdf.

[20] Ouzzani M, Hammady H, Fedorowicz Z, Elmagarmid A (2016). Rayyan—a web and mobile app for systematic reviews. Syst Rev.

[21] McHugh ML (2012). Interrater reliability: the kappa statistic. Biochem Med.

[22] Hong QN, Gonzalez-Reyes A, Pluye P (2018). Improving the usefulness of a tool for appraising the quality of qualitative, quantitative and mixed methods studies, the mixed methods Appraisal Tool (MMAT). J Eval Clin Pract.

[23] Agarwal S, Raymond JK, Schutta MH, Cardillo S, Miller VA, Long JA (2017). An Adult Health Care–Based Pediatric to Adult Transition Program for emerging adults with type 1 diabetes. Diabetes Educ.

[24] Colver A, McConachie H, Le Couteur A, Dovey-Pearce G, Mann KD, McDonagh JE (2018). A longitudinal, observational study of the features of transitional healthcare associated with better outcomes for young people with long-term conditions. BMC Med.

[25] Lyons SK, Ebekozien O, Garrity A, Buckingham D, Odugbesan O, Thomas S (2021). Increasing insulin pump use among 12- to 26-Year-Olds with type 1 diabetes: results from the T1D Exchange Quality Improvement Collaborative. Clin Diabetes.

[26] Pyatak EAPD, Sequeira PAMD, Vigen CLPPD, Weigensberg MJMD, Wood JRMD, Montoya L (2016). Clinical and psychosocial outcomes of a structured transition program among young adults with type 1 diabetes. J Adolesc Health.

[27] Raymond JK, Berget CL, Driscoll KA, Ketchum K, Cain C (2016). Fred” Thomas JF. CoYoT1 clinic: innovative telemedicine care model for young adults with type 1 diabetes. Diabetes Technol Ther.

[28] Reid MW, Krishnan S, Berget C, Cain C, Thomas JF, Klingensmith GJ (2018). CoYoT1 clinic: home telemedicine increases young adult engagement in diabetes care. Diabetes Technol Ther.

[29] Farrell K, Fernandez R, Salamonson Y, Griffiths R, Holmes-Walker DJ (2018). Health outcomes for youth with type 1 diabetes at 18 months and 30 months post transition from pediatric to adult care. Diabetes Res Clin Pract.

[30] Bisno DI, Reid MW, Fogel JL, Pyatak EA, Majidi S, Raymond JK. Virtual Group appointments reduce distress and improve Care Management in Young adults with type 1 diabetes. J Diabetes Sci Technol. 2021:19322968211035768. 10.1177/19322968211035768 PMC9631532 34328029

[31] Egan EA, Corrigan J, Shurpin K (2015). Building the Bridge from Pediatric to Adult Diabetes Care: making the connection. Diabetes Educ.

[32] Carey K, Morgan JR, Lin MY, Kain MS, Creevy WR (2020). Patient outcomes following total joint replacement surgery: a comparison of hospitals and ambulatory surgery Centers. J Arthroplasty.

[33] Sequeira PA, Pyatak EA, Weigensberg MJ, Vigen CP, Wood JR, Ruelas V (2015). Let’s Empower and prepare (LEAP): evaluation of a structured transition program for young adults with type 1 diabetes. Diabetes Care.

[34] Rueter P, Farrell K, Phelan H, Colman P, Craig ME, Gunton J (2021). Benchmarking care outcomes for young adults with type 1 diabetes in Australia after transition to adult care. Diabetes Metab J.

[35] Price CS, Corbett S, Lewis-Barned N, Morgan J, Oliver LE, Dovey-Pearce G (2011). Implementing a transition pathway in diabetes: a qualitative study of the experiences and suggestions of young people with diabetes. Child Care Health Dev.

[36] Peeters MAC, Sattoe JNT, Bronner MB, Bal RA, van Staa A. The added value of transition programs in dutch diabetes care: a controlled evaluation study. J Pediatr Nurs. 2021. 10.1016/j.pedn.2021.08.004 34419327

[37] Schmidt S, Markwart H, Bomba F, Muehlan H, Findeisen A, Kohl M (2018). Differential effect of a patient-education transition intervention in adolescents with IBD vs. diabetes. Eur J Pediatr.

[38] Grol R, Grimshaw J (2003). From best evidence to best practice: effective implementation of change in patients’ care. Lancet.

[39] Wensing M, Grol R (2019). Knowledge translation in health: how implementation science could contribute more. BMC Med.

[40] Altman L, Zurynski Y, Breen C, Hoffmann T, Woolfenden S (2018). A qualitative study of health care providers’ perceptions and experiences of working together to care for children with medical complexity (CMC). BMC Health Serv Res.

[41] Zurynski Y, Ellis LA, Dammery G, Smith CL, Halim N, Ansell J, Gillespie J, Caffery L, Vitangcol K, Wells LB. J. The Voice of Australian Health Consumers: The 2021 Australian Health Consumer Sentiment Survey. Report prepared for the Consumers Health Forum of Australia, 2022. ISBN: 978-1-74138-491-8. 2021.

[42] Shelton RC, Cooper BR, Stirman SW (2018). The sustainability of evidence-based interventions and practices in public health and health care. Annu Rev of Public Health.

[43] Samarasinghe SC, Medlow S, Ho J, Steinbeck K. Chronic illness and transition from paediatric to adult care: a systematic review of illness specific clinical guidelines for transition in chronic illnesses that require specialist to specialist transfer. J Transit Med. 2020;2(1).

[44] The Sydney Children’s Hospitals Network, Trapeze. Available from: http://www.trapeze.org.au/ 2021.

[45] Paone MC, Wigle M, Saewyc E (2006). The ON TRAC model for transitional care of adolescents. Prog Transpl.

[46] Damschroder LJ, Reardon CM, Widerquist MAO, Lowery J (2022). The updated Consolidated Framework for implementation research based on user feedback. Implement Sci.

[47] Damschroder LJ, Aron DC, Keith RE, Kirsh SR, Alexander JA, Lowery JC (2009). Fostering implementation of health services research findings into practice: a consolidated framework for advancing implementation science. Implement Sci.

[48] Shea CM, Jacobs SR, Esserman DA, Bruce K, Weiner BJ (2014). Organizational readiness for implementing change: a psychometric assessment of a new measure. Implement Sci.

