# Supplementary material for: Transition models of care for type 1 diabetes: a systematic review
Source: BMC Health Serv Res. 2023 Jul 20;23:779. doi: 10.1186/s12913-023-09644-9 (PMC10360292; doi:10.1186/s12913-023-09644-9)
Supplement: Supplementary file 1 — Supplementary Material 1 [file 12913_2023_9644_MOESM1_ESM.docx]

Supplemental File 1. Search strategies for the four databases (Scopus, Medline, CINAHL, and EMBASE)

| # | Query | 06/06/21 | 20/1/22 |
| --- | --- | --- | --- |

| 1 | Diabetes Mellitus, Type 1/ or diabetes mellitus/ | 196,238 | 204,477 |
| --- | --- | --- | --- |
| 2 | (iddm or insulin dependent diabetes mellitus or insulin-dependent mellitus or type 1 diabetes or diabetes type 1).mp. | 61,410 | 63738 |
| 3 | 1 or 2 | 216,611 | 224722 |
| 4 | infant/ or child/ or adolescent/ or young adult/ | 3,652,085 | 3,784,526 |
| 5 | (child* or infant* or teen* or adolescen* or young adult*).mp. | 3,856,033 | 3,987,479 |
| 6 | 4 or 5 | 4,573,213 | 4, 715421 |
| 7 | Clinical pathway/ or intervention study/ or evaluation study/ | 792,713 | 800,112 |
| 8 | (model* of care or care model* or clinic* pathway* or referral pathway*).mp. | 20,888 | 22,385 |
| 9 | (model* adj2 (service* or care)).ti,ab. | 23,034 | 24,646 |
| 10 | "delivery of health care"/ | 96,755 | 104,233 |
| 11 | (service* adj2 (initiativ* or configurat* or deliver* or capabilit*)).tw. | 22,372 | 23,893 |
| 12 | (intervention* adj2 (target* or service* or strateg*)).tw. | 59,248 | 63,972 |
| 13 | (service* adj2 (framework* or infrastructure)).tw. | 1,806 | 1,914 |
| 14 | or/7-13 | 985,968 | 1,008,105 |
| 15 | "delivery of health care, integrated"/ or transitional care/ or patient education/ or transition to adult care/ or Treatment Outcome/ or Outcome Assessment, Health Care/ | 1,191,939 | 1,247,513 |
| 16 | (transitional care or care transitions or integrated care or multidisciplinary care or patient-centered care or transition to adult care or shared care plan or team-based care or team care or diabetes education or multidisciplinary team* or interdisciplinary care* or outcome*).mp. | 2,715,410 | 2,873,827 |
| 17 | 15 or 16 | 2,791,312 | 2,950,462 |
| 18 | 3 and 6 and 14 and 17 | 791 | 622 |
| 19 | limit 18 to (english language and yr="2010 - 2022") | 395 | 426 |
| 20 | limit to( yr="2021") |  | 28 |
