# Supplementary material for: Transition models of care for type 1 diabetes: a systematic review
Source: BMC Health Serv Res. 2023 Jul 20;23:779. doi: 10.1186/s12913-023-09644-9 (PMC10360292; doi:10.1186/s12913-023-09644-9)
Supplement: Supplementary file 2 — Supplementary Material 2 [file 12913_2023_9644_MOESM2_ESM.docx]

Supplemental file 2. Quality Appraisal of fourteen transitional studies using the Mixed Methods Appraisal Tool (MMAT)

| Reference | Screening Questions | | Qualitative Studies | | | | |
| --- | --- | --- | --- | --- | --- | --- | --- |
|  | Clear research question | Appropriate data to answer research question | Appropriate approach | Adequate data collection | Findings derived from data | Substantiated interpretation | Coherence between data collection, analysis and interpretation |
| Price et al. 2011^33^ | Y | Y | Y | Y | Y | Y | Y |
|  |  |  | Quantitative Randomised Controlled Trials | | | | |
|  |  |  | Appropriate randomisation | Comparable groups at baseline | Complete outcome data | Blinded outcome assessors | Adherence to intervention |
| Bisno et al. 2021^30^ | Y | Y | Y | Y | Y | N | Y |
|  |  |  | Quantitative Non-Randomised | | | | |
|  |  |  | Are participants representative | Appropriate measurements | Complete outcome data | Confounders accounted for | Intervention administered as intended |
| Agarwal et al. 2017^23^ | Y | Y | Y | Y | Y | Y | Y |
| Colver et al. 2018^24^ | Y | Y | Y | Y | Y | Y | Y |
| Lyons et al. 2021^25^ | Y | Y | Y | Y | N | N | Y |
| Pyatak et al. 2017^26^ | Y | Y | Y | Y | Y | Y | Y |
| Raymond et al. 2016^27^ | Y | Y | Y | Y | Y | N | Y |
| Reid et al. 2018^28^ | Y | Y | Y | Y | Y | Y | Y |
| Rueter et al. 2021^35^ | Y | Y | Y | Y | Y | Y | Y |
| Schmidt et al. 2018^37^ | Y | Y | Y | Y | Y | Y | Y |
| Sequiera et al. 2015^34^ | Y | Y | Y | Y | Y | U | Y |
|  |  |  | Quantitative Descriptive | | | | |
|  |  |  | Sampling strategy relevant to address the RQ? | Sample representative of the target population? | Appropriate measurements? | Low risk of non-response bias? | Appropriate statistical analysis? |
| Farrell et al. 2018^29^ | Y | Y | Y | Y | Y | Y | Y |
|  |  |  | Mixed Methods | | | | |
|  |  |  | Adequate rationale for MM approach | Components effectively integrated to answer research question | Outputs adequately interpreted | Divergences adequately addressed | Adherence to quality criteria for each component |
| Egan et al. 2015^31^ | Y | Y | Y | Y | Y | Y | Y |
| Peeters et al. 2021^36^ | Y | Y | Y | Y | Y | Y | Y |
